# Migraine Features and Nociceptive Profile Do Not Influence Conditioned Pain Modulation in Women With Episodic and Chronic Migraine

**DOI:** 10.1155/prm/8331529

**Published:** 2026-05-08

**Authors:** Ana I. de-la-Llave-Rincón, Silvia Ambite-Quesada, Juan C. Pacho-Hernández, Angela Tejera-Alonso, Cristina Gómez-Calero, César Fernández-de-las-Peñas, Beatriz Alvarez-Mariño, Lars Arendt-Nielsen, Margarita Cigarán-Méndez

**Affiliations:** ^1^ Department of Physical Therapy, Occupational Therapy, Physical Medicine and Rehabilitation, Rey Juan Carlos University, Madrid, Spain, urjc.es; ^2^ Department of Research and Psychology on Education, Complutense de Madrid University, Madrid, Spain; ^3^ Department of Psychology, Rey Juan Carlos University, Madrid, Spain, urjc.es; ^4^ Department of Health Science and Technology, Center for Neuroplasticity and Pain (CNAP), Faculty of Medicine, Aalborg University, Aalborg, Denmark, aau.dk; ^5^ Department of Neurology, Rey Juan Carlos Hospital, Móstoles, Spain; ^6^ Department of Gastroenterology & Hepatology, Mech-Sense, Clinical Institute, Aalborg University Hospital, Aalborg, Denmark, aalborguh.rn.dk; ^7^ Steno Diabetes Center North Denmark, Clinical Institute, Aalborg University Hospital, Aalborg, Denmark, aalborguh.rn.dk

**Keywords:** conditioned pain modulation, disability, migraine, neuropathic, sensitization

## Abstract

**Objective:**

To determine the influence of sensitization‐associated and neuropathic‐like symptoms and clinical features in conditioned pain modulation (CPM) activity in women with episodic or chronic migraine.

**Methods:**

A cross‐sectional study including 70 women with chronic migraine and 70 with episodic migraine was performed. Clinical migraine features (related disability, pain intensity, frequency, and duration) and pain processing variables (sensitization‐associated or neuropathic‐like symptoms) were evaluated with self‐reported outcomes. Pressure pain thresholds (PPTs) at the temporalis muscle, lateral epicondyle, and tibialis anterior were bilaterally assessed. Heat (HPT) and cold (CPT) pain thresholds at the frontalis (V1 area) muscle were also assessed. Subsequently, CPM was evaluated immediately after a 1‐min cold‐pressor test paradigm on changes obtained in PPTs, HPT, and CPT.

**Results:**

No significant group ∗ time interactions after controlling for migraine features, related disability (MIDAS, HIT‐6), sensitization‐associated (CSI), or neuropathic‐like symptoms (S‐LANSS) were found for PPTs at the temporalis muscle (Wilk’s *λ* = 0.996, *F*[1128] = 0.500, *p* = 0.481, *n* ^2^ *p* = 0.004, 1 − *β* = 0.108), lateral epicondyle (Wilk’s *λ* = 0.997, *F*[1128] = 0.369, *p* = 0.545, *n* ^2^ *p* = 0.003, 1 − *β* = 0.093), and tibialis anterior (Wilk’s *λ* = 0.999, *F*[1128] = 0.070, *p* = 0.792, *n* ^2^ *p* = 0.001, 1 − *β* = 0.058) as well as CPT (Wilk’s *λ* = 0.991, *F*[1128] = 1.136, *p* = 0.288, *n* ^2^ *p* = 0.009, 1 − *β* = 0.185) or HPT (Wilk’s *λ* = 0.992, *F*[1128] = 0.992, *p* = 0.315, *n* ^2^ *p* = 0.008, 1 − *β* = 0.170): no significant changes in PPTs, CPTs, or HPTs were seen after the conditioned stimulus in women with either episodic or chronic migraine. No significant effect of clinical or pain processing outcomes on CPM was seen.

**Conclusion:**

This study showed similar CPM activation (lack of) in women with episodic and chronic migraine. Thus, clinical features and the presence of sensitization‐associated or neuropathic‐like symptoms did not modulate CPM activity in women with migraine.

## 1. Introduction

Migraine is a primary headache showing a one‐year prevalence of 15% in the general adult population with a prevalence peak in people aged 35–39 years [1]. Migraine is the leading cause of disability in people younger than 50 years old, particularly women [2], and it causes social, work, economic, and health impacts on the society [3].

Several hypotheses, for example, sensitive, vascular, or cortical, have been proposed to explain migraine [4]. The sensitive hypothesis suggests peripheral/central sensitization processes as mechanisms involved in migraine. A sensitized state can be characterized by exaggerated responses (facilitation) to different stimuli (hyperalgesia and allodynia) and/or the presence of impaired conditioned pain modulation (CPM), that is, inhibition of descending inhibitory pathways [5]. Current evidence supports the presence of hyperalgesia to pressure, thermal, or electrical stimuli within the trigeminal area in people with migraine, but the presence of hyperalgesia in extra‐trigeminal, distant areas (suggestive of central sensitization) is still controversial [6, 7]. Thus, evidence about the presence of deficits in CPM in migraine is heterogeneous. Some studies reported impaired CPM in people with migraine, while others did not find such a deficit when compared with healthy controls [8, 9]. These reviews concluded that the observed conflicting results could be related to (1) large variability in those CPM paradigms used; (2) small sample sizes including both sexes (males/females); (3) no differentiation between episodic/chronic migraine; and (4) no control of factors affecting pain processing [8, 9].

It should be noted that most studies investigating CPM activation in individuals with migraine have not considered either the presence of sensitization‐associated symptoms or clinical features related to migraine. Patient‐reported outcome measures (PROMs) consist of self‐reported questionnaires assessing different aspects of any condition. The Central Sensitization Inventory (CSI) [10] and the Self‐administered Leeds Assessment of Neuropathic Symptoms and Signs (S‐LANSS) [11] are the PROMs commonly used for assessing the presence of sensitization‐associated or neuropathic‐like symptoms, respectively. The use of the CSI revealed that 20%–30% of patients with migraine reported sensitization‐associated symptomatology [12]. In addition, it has been suggested that migraine can share common mechanisms with neuropathic pain [13]; however, no previous study has used the S‐LANSS to identify the presence of self‐rated neuropathic‐like symptoms in migraine. Studies investigating CPM activity in patients with migraine, controlling the influence of pain processing symptomatology, are clearly needed.

Since pain processing mechanisms are different between men and women [14] and since migraine is more prevalent in women [1], clinical research should be conducted with a “sex perspective” [15]. Therefore, in the current study we exclusively included women with migraine. Accordingly, the aim of our study was to determine the influence of sensitization‐associated and neuropathic‐like symptoms on CPM activation in women with migraine, attending to their chronicity and clinical features.

## 2. Methods

### 2.1. Participants

A cross‐sectional observational study following the Strengthening the Reporting of Observational Studies in Epidemiology (STROBE) guidelines [16] was performed. The study design was approved by Local Ethics Committees of the institutions involved (HUFA 24_117; URJC_010220240912024). All participants were informed and signed the informed consent before their inclusion in the study.

Consecutive women attending the headache the unit at neurology department of Hospital Universitario Fundación Alcorcón (HUFA), an urban hospital in Madrid (Spain), were eligible to participate. To be potentially included, they should present a migraine diagnosed according to the third edition (ICHD‐III) of the International Headache Society (IHS) [17] by an experienced neurologist. Migraine history included location, quality of pain, years with migraine, family history, and medication intake. They were excluded if present: (1) other primary and/or secondary headache [17]; (2) history of cervical trauma (i.e., whiplash) or herniated disk; (3) comorbid medical diseases (e.g., fibromyalgia); (4) any psychiatric diagnosis; (5) taking medication (e.g., antipsychotics, anticonvulsants, or anticholinergics) affecting cognition; (6) pregnancy; or (7) had received any medical intervention, including anesthetic blocks, in the past 3 months.

### 2.2. Patient‐Related Outcome Measures (PROMs) Related to Migraine

Migraine clinical data were collected into a headache diary for 4 weeks, where patients registered the frequency of migraine attacks (days/month), the duration of each migraine attack (hours), and the intensity of each attack (numerical pain rate scale [NPRS], 0–10 points) [18].

Migraine‐related disability was evaluated with the Migraine Disability Assessment Scale (MIDAS) and the 6‐item Headache Impact Test (HIT‐6). The MIDAS evaluates disability by asking about the days of partial or total loss within the previous 3 months caused by migraine regarding (1) paid work (or school); (2) household chores; and (3) family, social, and leisure activities [19]. The score consists of the sum of missed days and classifies migraine‐related disability as little/no disability (score 0–5), mild disability (score 6–10), moderate disability (score 11–20), or severe disability (score ≥ 21) [20]. The Spanish version of the MIDAS questionnaire is a valid and reliable tool to measure migraine‐related disability [21].

The HIT‐6 items questionnaire assesses the adverse impact of headaches by asking five questions (on a 5‐point Likert scale) related to social functioning, role functioning, vitality, cognitive functioning, and psychological distress. The total score ranges from 36 to 78 points, where higher scores are associated with a higher impact of migraine. The HIT‐6 has good psychometric properties in migraine [22] and has been validated in the Spanish language [23].

### 2.3. PROMs Related to Pain Processing

The CSI and the S‐LANSS were PROMs used as proxies to evaluate symptomatology associated with pain processing. The Spanish version of the CSI was used to identify the presence of sensitization‐associated symptoms [24]. Each symptom is scored on a 5‐point Likert scale, providing a score ranging from 0 to 100 points, where a cutoff score of 40 points suggests the presence of sensitization‐associated symptoms [25]. The CSI has shown good psychometric properties for assessing sensitization‐associated symptoms in individuals with persistent chronic pain [26]. The Spanish version of S‐LANSS was used to assess the presence of neuropathic‐like associated pain symptomatology [27]. The S‐LANSS uses seven questions providing a score ranging from 0 to 24 points, where a cutoff score of ≥ 12 points is indicative of the presence of neuropathic‐like pain symptoms [11].

### 2.4. CPM

In women with episodic migraine, the evaluation was conducted when they were headache‐free and when at least 1 week had elapsed since the last headache to avoid migraine‐related allodynia. In those with chronic migraines, the evaluation was conducted pain‐free (if possible) or when the intensity of the pain was < 3 points. Thus, evaluation was tried to be at least one to two days since the last headache. All participants were asked to avoid taking symptomatic medication, including analgesics or muscle relaxants, at least 48 h prior to the examination. No change in their prophylactic treatment was made.

The CPM paradigm used was the cold‐pressor test [28], which has shown good to excellent reliability when used in chronic pain [29]. The first two stimuli (mechanical and thermal) were applied in random order, then the patient was exposed to the conditioned stimulus for 1 min, and, finally, both stimuli were applied again in random order immediately after the hand was withdrawn from the cold water. Pressure pain thresholds (PPTs) were assessed bilaterally at a trigeminal area (the temporalis muscle) and two remote pain‐free areas (lateral epicondyle and tibialis anterior muscle) with an electronic algometer (Somedic AB, Farsta, Sweden). Pressure was applied at a rate of approximately 30 kPa/s with a random order in the point. Three repetitions were made at each point with an interval of 30 s to avoid temporal summation; the mean was calculated and used for statistical analyses.

Heat (HPT) and cold (CPT) pain thresholds were bilaterally calculated on the frontalis muscle (innervated by the first trigeminal branch) using an advanced thermal stimulator (ATS System, Medoc Pathways System, Israel) following the method of limits [30]. Three repetitions were obtained with an interval of 30 s; the mean was calculated and used for statistical analyses.

Second, participants immersed one hand (the hand on the side of the migraine in women with unilateral migraine and the dominant hand in women with bilateral migraine) for one minute in a water container (Huber K20‐cc NR, Offenburg, Germany) at a temperature of 10°C. Since attentional focus on the conditioned stimulus reduces CPM efficacy [31], it is recommended to not rate the intensity of induced pain during the conditioned stimulus application to prevent a reduction of the inhibitory effect. Finally, participants were asked to remove their hand from water, and both mechanical and thermal stimuli were assessed again following the same procedure described above.

An impaired CPM is operationally defined as no change or even a negative change in data obtained before and after the conditioned stimulus [32]. We calculated the absolute (kPa or °C, CPM change score) and the percentage (%, CPM activation index) differences between PPT, HPT and CPT values before and after the conditioned stimulus.

### 2.5. Sample Size Calculation

The estimated sample size was calculated using the G∗Power 3.1.9.7 computer program. The following statistical parameters were entered into the “F tests” for repeated‐measures MANOVA statistical test on the main outcome of the study, CPM: effect size (f2): 0.25; *α*: 0.05; statistical power: 0.80; number of groups: 2; number of measures: 2. According to these parameters, the necessary sample size identified was at least 120 subjects.

### 2.6. Statistical Analysis

All statistical analyses were conducted using SPSS statistical software. Outliers were identified through boxplots. Assumptions of normality and sphericity were verified. Means (standard deviations) are provided for continuous variables, whereas frequencies (percentages) are provided for categorical variables. Chi‐square (categorical variables) and Student’s *t*‐tests (for continuous variables) were applied to calculate between‐group differences (episodic or chronic migraine) to identify potential covariates to be included in posterior analyses and to identify between‐group differences for pain profile outcomes. Thus, the association between pain profile outcomes (e.g., CSI, S‐LANSS, PPTs, HPT, and CPT) and clinical migraine features was evaluated with Pearson correlation coefficients (r) on each migraine group.

The primary analysis included separate multivariate repeated‐measures analysis of covariance (OMNIBUS RM‐ANOVA) to identify the effect of migraine on CPM (change in PPT, HPT, and CPT) by controlling potential confounders significantly different between groups. For these analyses, psychophysical (PPT, HPT, and CPT) outcomes (before/after the conditioning stimulus) were the within‐subject factor; group (episodic migraine and chronic migraine) was the independent variable; and clinical and pain processing variables were the covariates. We applied a correction for multiple comparisons, where *p* values < 0.025 (0.05/2) were considered statistically significant. Partial eta squared (*n* ^2^ *p*) was finally calculated to determine effect sizes, with values of 0.01 indicating a small effect, 0.06 a medium effect, and above 0.14 a large effect [33].

## 3. Results

### 3.1. Descriptive Data and Intergroup Comparisons

Clinical and related‐disability migraine data, as well as pain processing outcomes of women with episodic or chronic migraine, are shown in Table 1. Significant between‐groups differences in pain intensity (*p* < 0.001), frequency (*p* < 0.001), and duration (*p* = 0.040) of migraine, MIDAS (*p* = 0.009), CSI (*p* = 0.010), PPT at the lateral epicondyle (*p* = 0.014), and HPT (*p* = 0.011) were found: women with chronic migraine reported higher intensity (mean difference: 0.95 points, 95% CI 0.45 to 1.45), frequency (mean difference: 13.0 days/months, 95% CI 11.4–14.6), and duration (mean difference: 7.8 h/attack, 95% CI 0.4–15.25) of migraine, higher MIDAS (mean difference: 32.1 points, 95% CI 8.5 to 55.7, *p* = 0.009), and CSI (mean difference: 6 points, 95% CI 1.45 to 10.55, *p* = 0.01) scores as well as lower PPTs at the lateral epicondyle (mean difference: −64.3 kPa, 95% CI −103.6 to −25.0) and HPT (mean difference: −2.7, 95% CI −3.3 to −2.1) than women with episodic migraine. A significantly higher proportion of women with chronic migraine self‐reported sensitization‐associated symptomatology as determined by the CSI score (*p* < 0.001).

**TABLE 1:** Clinical features and pain processing variables of women with episodic or chronic migraine.

	Chronic migraine (*n* = 70)	Episodic migraine (*n* = 70)	*t*	*p*
	Mean (SD)	Mean (SD)		
Age (years)	46.1 (5.2)	48.1 (7.2)	1.859	0.065
*Clinical and related-disability outcomes*				
Migraine intensity (NPRS, 0–10)	8.6 (1.4)	7.6 (1.55)	−3.766	0.001
Migraine frequency (days per month)	16.5 (6.4)	3.5 (2.7)	−15.760	< 0.001
Migraine duration (hours per attack)	33.1 (20.3)	25.3 (23.9)	−2.078	0.040
Migraine impact (HIT‐6, 36–78)	61.3 (7.9)	61.7 (7.3)	0.255	0.799
Migraine‐related disability (MIDAS)	62.3 (96.2)	31.2 (30.0)	−2.667	0.009
Preventive treatment				
Yes (amitriptyline)	52 (74.3%)	48 (68.6%)	0.560 (*χ* ^2^)	0.454
No	18 (25.7%)	22 (31.4%)		
Symptomatic treatment				
Yes	59 (84.3%)	62 (88.6%)	0.548 (*χ* ^2^)	0.459
No	11 (15.7%)	8 (11.4%)		
Type of symptomatic treatment				
Painkillers	5 (8.5%)	4 (6.5%)	0.180 (*χ* ^2^)	0.672
NSAIDs	54 (91.5%)	58 (93.5%)		

*Pain processing outcomes*				
S‐LANSS score (0–24)	13.5 (6.9)	14.0 (6.7)	0.483	0.630
Neuropathic‐like symptoms (S‐LANSS ≥ 12), *n* (%)	46 (65.7%)	44 (62.9%)	0.124 (*χ* ^2^)	0.724
CSI score (0–100)	51.4 (14.4)	45.4 (12.8)	2.599	0.010
Sensitization‐associated symptoms (CSI ≥ 40), *n* (%)	58 (82.90%)	37 (52.90%)	14.442 (*χ* ^2^)	< 0.001
Pressure pain thresholds (PPT, kPa)				
Temporalis muscle	286.0 (13.1)	284.9 (13.3)	−0.105	0.965
Lateral epicondyle	299.7 (14.5)	364.0 (14.6)	2.570	0.014
Tibialis anterior	351.1 (13.2)	375.7 (13.3)	1.051	0.295
Thermal pain thresholds (°C)				
Cold pain thresholds (CPT)	18.0 (0.85)	15.2 (0.9)	−1.901	0.063
Heat pain threshold (HPT)	41.0 (0.6)	43.7 (0.6)	2.931	0.011

*Note: n*: number of subjects, HIT‐6: 6‐item Headache Impact Test; MIDAS: Migraine Disability Assessment Scale; S‐LANSS: Self‐administered Leeds Assessment of Neuropathic Symptoms and Signs.

Abbreviations: CSI, central sensitization inventory; SD, standard deviation.

### 3.2. Association Between Pain Profile and Clinical Migraine Outcomes

In women with chronic migraine, significant positive associations between CSI score and migraine intensity (*r* = 0.393, *p* = 0.001), HIT‐6 (*r* = 0.511, *p* < 0.001), MIDAS (*r* = 0.351, *p* = 0.003) and the S‐LANSS (*r* = 0.654, *p* < 0.001) were observed. Thus, the S‐LANSS score was also significantly and positively associated with migraine intensity (*r* = 0.236, *p* = 0.049), HIT‐6 (*r* = 0.467, *p* < 0.001), and MIDAS (*r* = 0.415, *p* < 0.001). No association between PROMs of pain processing, for example, CSI or S‐LANSS, clinical and related‐disability features of migraine, and psychophysical outcomes, for example, PPTs, CPT or HPT, were identified.

In women with episodic migraine, significant positive associations between CSI score and migraine intensity (*r* = 0.261, *p* = 0.035), HIT‐6 (*r* = 0.553, *p* < 0.001), MIDAS (*r* = 0.326, *p* = 0.009) and the S‐LANSS (*r* = 0.368, *p* = 0.003) were observed. Thus, the S‐LANSS score was also significantly and positively associated with the HIT‐6 score (*r* = 0.261, *p* = 0.035). No association between PROMs of pain processing, for example, CSI or S‐LANSS, clinical and related‐disability features of migraine, and psychophysical outcomes, for example, PPTs, CPT or HPT, were identified.

### 3.3. Mechanical CPM in the Trigeminal Area (PPTs Temporalis Muscle)

The repeated‐measures MANCOVA analyses (the estimated marginal means and standard deviations are shown in Table 2) did not reveal a significant time effect for PPTs (Wilk’s *λ* = 0.985, *F*[1128] = 1.895, *p* = 0.171, *n* ^2^ *p* = 0.015, 1 − *β* = 0.277) nor a significant group ∗ time interaction effect (Wilk’s *λ* = 0.996, *F*[1128] = 0.500, *p* = 0.481, *n* ^2^ *p* = 0.004, 1 − *β* = 0.108) after controlling for migraine intensity, frequency, duration, related disability, sensitization‐associated, and neuropathic‐like symptoms. Thus, the mechanical CPM paradigm in the trigeminal area did not reveal a significant decrease (*p* = 0.641) in CPM activation index for women with episodic (mean −1.85%, SD 0.1%) or chronic (mean −3.0%, SD 0.1%) migraine.

**TABLE 2:** Pressure pain thresholds (PPTs, kPa) before and after the conditioned stimulus (cold‐pressor test) in the temporalis muscle.

	**Chronic migraine (*n* = 70)**	**Episodic migraine (*n* = 70)**	**Univariate tests**			
**Intergroup differences**						
**PPT values**	**Mean (SD)**	**Mean (SD)**	***F***	**p**	**η** ^2^ **p**	**1 − *β***

Before	286.0 (13.1)	284.9 (13.3)	0.002	0.965	0.000	0.050
After	282.3 (14.2)	274.1 (14.3)	0.106	0.745	0.001	0.062

**Intragroup differences**						
	**Before conditioned stimulus**	**After conditioned stimulus**				
**Group**	**Mean (SD)**	**Mean (SD)**	***F***	**p**	**η** ^2^ **p**	**1 − *β***

Chronic migraine	286.0 (13.1)	282.3 (14.2)	0.423	0.517	0.003	0.099
Episodic migraine	284.9 (13.3)	274.1 (14.3)	3.560	0.061	0.027	0.465

*Note: n*: number of subjects.

Abbreviation: SD, standard deviation.

### 3.4. Mechanical CPM in Extra‐Trigeminal Area (PPTs Lateral Epicondyle)

The repeated‐measures MANCOVA (the estimated marginal means and standard deviations can be shown in Table 3) did not show a significant time effect for PPTs (Wilk’s *λ* = 1.000, *F*[1128] = 0.015, *p* = 0.903, *n* ^2^ *p* = 0.000, 1 − *β* = 0.052) nor a group ∗ time interaction effect (Wilk’s *λ* = 0.997, *F*[1128] = 0.369, *p* = 0.545, *n* ^2^ *p* = 0.003, 1 − *β* = 0.093) after controlling for migraine intensity, frequency, duration, related disability, sensitization‐associated, and neuropathic‐like symptoms. The mechanical CPM paradigm in the extra‐trigeminal area did not show a significant decrease (*p* = 0.100) in CPM activation index for women with episodic (mean −3.8%, SD 0.12%) or chronic (mean −7.6%, SD 0.14%) migraine.

**TABLE 3:** Pressure pain thresholds (PPTs, kPa) before and after the conditioned stimulus (cold‐pressor test) in the lateral epicondyle.

	**Chronic migraine (*n* = 70)**	**Episodic migraine (*n* = 70)**	**Univariate tests**			
**Intergroup differences**						
**PPT values**	**Mean (SD)**	**Mean (SD)**	***F***	**p**	**η** ^2^ **p**	**1 − *β***

Before	299.7 (14.5)	364.0 (14.6)	6.252	0.014	0.047	0.699
After	285.4 (15.5)	342.8 (15.7)	4.290	0.040	0.032	0.538

**Intragroup differences**						
	**Before conditioned stimulus**	**After conditioned stimulus**				
**Group**	**Mean (SD)**	**Mean (SD)**	***F***	**p**	**η** ^2^ **p**	**1 − *β***

Chronic migraine	299.7 (14.5)	285.4 (15.5)	5.028	0.027	0.038	0.605
Episodic migraine	364.0 (14.6)	342.8 (15.7)	10.800	0.001	0.078	0.903

*Note: n*: number of subjects.

Abbreviation: SD, standard deviation.

### 3.5. Mechanical CPM in Distant Area (PPTs Tibialis Anterior Muscle)

The repeated‐measures MANCOVA analyses (the estimated marginal means and standard deviations are shown in Table 4) did not find a significant main effect of time for PPTs (Wilk’s *λ* = 0.999, *F*[1128] = 0.126, *p* = 0.723, *n* ^2^ *p* = 0.001, 1 − *β* = 0.064) nor a significant group ∗ time interaction effect (Wilk’s *λ* = 0.999, *F*[1128] = 0.070, *p* = 0.792, *n* ^2^ *p* *p* = 0.001, 1 − *β* = 0.058) after controlling for migraine intensity, frequency, duration, related disability, sensitization‐associated symptoms, or neuropathic‐like symptoms. The mechanical CPM paradigm in a distant pain‐free area did not reveal a significant (*p* = 0.679) decrease in CPM activation index for women with episodic (mean −0.3%, SD 0.1%) or chronic (mean 0.55%, SD 0.13%) migraine.

**TABLE 4:** Pressure pain thresholds (PPTs, kPa) before and after the conditioned stimulus (cold‐pressor test) in the tibialis anterior muscle.

	**Chronic migraine (*n* = 70)**	**Episodic migraine (*n* = 70)**	**Univariate tests**			
**Intergroup differences**						
**PPT values**	**Mean (SD)**	**Mean (SD)**	***F***	**p**	**η** ^2^ **p**	**1 − *β***

Before	351.1 (13.2)	375.7 (13.3)	1.108	0.295	0.009	0.181
After	348.1 (14.1)	376.2 (14.3)	1.247	0.266	0.010	0.198

**Intragroup differences**						
	**Before conditioned stimulus**	**After conditioned stimulus**				
**Group**	**Mean (SD)**	**Mean (SD)**	***F***	**p**	**η** ^2^ **p**	**1 − *β***

Chronic migraine	351.1 (13.2)	348.1 (14.1)	0.161	0.689	0.001	0.068
Episodic migraine	375.7 (13.3)	376.2 (14.3)	0.005	0.946	0.000	0.051

*Note: n*: number of subjects.

Abbreviation: SD, standard deviation.

### 3.6. Thermal CPM in the Trigeminal Area (CPT and HPT Frontalis Muscle)

The repeated‐measures MANCOVA analyses did not find a significant time effect for CPT (Wilk’s *λ* = 1.000, *F*[1128] = 0.004, *p* = 0.948, *n* ^2^ *p* = 0.000, 1 − *β* = 0.050, Table 5) or HPTs (Wilk’s *λ* = 0.985, *F*[1128] = 1.902, *p* = 0.170, *n* ^2^ *p* = 0.015, 1 − *β* = 0.278, Table 6).

**TABLE 5:** Cold pain thresholds (CPT, °C) before and after the conditioned stimulus (cold‐pressor test) in the frontalis muscle.

	**Chronic migraine (*n* = 70)**	**Episodic migraine (*n* = 70)**	**Univariate tests**			
**Intergroup differences**						
**CPT values**	**Mean (SD)**	**Mean (SD)**	***F***	**p**	**η** ^2^ **p**	**1 − *β***

Before	18.0 (0.85)	15.2 (0.9)	3.509	0.063	0.027	0.460
After	17.3 (0.9)	15.5 (0.9)	1.424	0.235	0.011	0.220

**Intragroup differences**						
	**Before conditioned stimulus**	**After conditioned stimulus**				
**Group**	**Mean (SD)**	**Mean (SD)**	***F***	**p**	**η** ^2^ **p**	**1 − *β***

Chronic migraine	18.0 (0.85)	17.3 (0.9)	1.723	0.192	0.013	0.256
Episodic migraine	15.2 (0.9)	15.5 (0.9)	0.334	0.565	0.003	0.088

*Note: n*: number of subjects.

Abbreviation: SD, standard deviation.

**TABLE 6:** Heat pain thresholds (CPT, °C) before and after the conditioned stimulus (cold‐pressor test) in the frontalis muscle.

	**Chronic migraine (*n* = 70)**	**Episodic migraine (*n* = 70)**	**Univariate tests**			
**Intergroup differences**						
**HPT values**	**Mean (SD)**	**Mean (SD)**	***F***	**p**	**η** ^2^ **p**	**1 − *β***

Before	41.0 (0.6)	43.7 (0.6)	6.591	0.011	0.049	0.722
After	41.5 (0.6)	43.7 (0.55)	5.237	0.024	0.039	0.622

**Intragroup differences**						
	**Before conditioned stimulus**	**After conditioned stimulus**				
**Group**	**Mean (SD)**	**Mean (SD)**	***F***	**p**	**η** ^2^ **p**	**1 − *β***

Chronic migraine	41.0 (0.6)	41.5 (0.6)	3.045	0.083	0.023	0.410
Episodic migraine	43.7 (0.6)	43.7 (0.55)	0.003	0.960	0.000	0.050

*Note: n*: number of subjects.

Abbreviation: SD, standard deviation.

Similarly, no significant group ∗ time interaction effects for CPT (Wilk’s *λ* = 0.991, *F*[1128] = 1.136, *p* = 0.288, *n* ^2^ *p* = 0.009, 1 − *β* = 0.185) or HPT (Wilk’s *λ* = 0.992, *F*[1128] = 0.992, *p* = 0.315, *n* ^2^ *p* = 0.008, 1 − *β* = 0.170) after controlling for migraine intensity, frequency, duration, related‐disability, sensitization‐associated, or neuropathic‐like symptomatology were found. The thermal CPM paradigm in the trigeminal area did not reveal a significant (*p* = 0.208) increase in CPM activation index for women with episodic (mean 0.3%, SD 0.05%) or chronic (mean 1.13, SD 0.03%) migraine.

## 4. Discussion

This study revealed that women with episodic and chronic migraine overall exhibited similar pain profiles based on PROMs of nociceptive processing, PPTs, and CPM. Only PPT at the lateral epicondyle and HPT at the trigeminal area revealed higher sensitivity to pain. Thus, the CSI was the only PROM revealing a higher presence of sensitization‐associated symptomatology in women with chronic migraine as compared with those with episodic migraine. Our results revealed similar CPM efficiency (lack of) to pressure pain and thermal pain sensitivity in women with episodic or chronic migraine. Thus, migraine pain features and sensitization‐related or neuropathic‐like symptoms did not modulate CPM in women with migraine.

The results of our study suggest a lack of CPM activation in sensitivity to pressure and thermal pain in women with episodic or chronic pain since no changes or even negative values of the CPM activation index were identified; however, the lack of a control group does not permit confirmation of this. In fact, former literature on CPM in migraine is heterogeneous, but the overall conclusion of current reviews is no difference in CPM efficiency between patients with migraine and healthy controls [8, 9]. As previously commented, conflicting data on CPM in migraine has been attributed to small samples, lack of characterization of migraine features, and use of different CPM paradigms. In fact, the meta‐analysis by van Welie et al. concluded that pain profiling in migraine patients is heterogeneous and that better understanding requires differentiation among migraine subtypes [9].

The current study focused on the influence of clinical and pain processing features of migraine on CPM activity. In fact, most previous studies on CPM did not differentiate between the episodic or chronic forms of migraine. Current results showed that neither clinical migraine features (i.e., intensity, duration, and frequency) nor related‐disability outcomes (e.g., MIDAS or HIT‐6) influence the CPM index in women with migraine, albeit women with chronic migraine exhibited worse clinical variables than those with episodic migraine.

It is suggested that chronic migraine could exhibit a more pronounced altered pain processing than episodic migraine. Evidence supports the presence of hyperexcitability of the nervous system in individuals with migraines, episodic or chronic, when compared with pain‐free controls, by using objective and self‐reported outcomes [6, 7, 12]; however, a recent meta‐analysis found no significant differences in most quantitative sensory tests between episodic or chronic migraine [34]. In agreement with Cnockaert et al. [34], we did not find overall differences in pressure or thermal pain hyperalgesia between women with episodic or chronic migraine, only small differences in PPT at the lateral epicondyle and HPT in the trigeminal area. On the contrary, we observed that a greater proportion of women with chronic migraine self‐reported sensitization‐associated symptoms (CSI score > 40 points). Suzuki et al. concluded, from a narrative review, that 20%–30% of patients with migraine self‐reported sensitization‐associated symptoms according to the CSI score [12]. Our study observed higher prevalence rates of sensitization‐associated symptoms (52% in episodic, 82% in chronic) than previous studies [12]. The lower CSI scores (from 28 to 34 points) reported in studies included in the Suzuki et al. review [12] could explain these discrepancies. Barone et al. have recently reported a CSI score (mean: 41, SD: 13 points) [35] similar to scores found in our study. Population‐based studies with a better characterization of self‐reported sensitization‐associated symptoms in the general migraine population are needed. Accordingly, our results support the presence of sensitization‐associated symptoms but similar CPM activity in women with chronic migraine. Thus, CSI was not associated with CPM activity in episodic or chronic migraine groups. The fact that most women with migraine did not exhibit changes in the CPM index could explain the lack of an association between both outcomes. It is possible that both outcomes, for example, CSI and CPM, represent two different aspects of altered nociceptive pain processing. In addition, we did not observe significant associations between psychophysical outcomes, that is, PPTs, HPT or CPT, and clinical features of migraine or pain processing PROMs in women with either episodic or chronic migraine. This lack of association agrees with previous data suggesting no linear associations between PPT and clinical and related‐disability variables in different chronic pain conditions [36]. Previous and current data would support the headache continuum hypothesis, where episodic and chronic migraine could share similar altered nociceptive processing [37].

In addition, it has been suggested that migraine can share common mechanisms with neuropathic pain [13]; however, no previous study has investigated the presence of self‐reported neuropathic‐like pain symptoms in this population. We observed that up to 60% of our sample of women with migraine exhibited neuropathic‐like pain symptomatology as expressed by the S‐LANSS scores > 12 points. Interestingly, cutaneous allodynia, a sign commonly used for identifying neuropathic pain, is also present in 60%–70% of patients with migraine [38]. Cutaneous allodynia is a marker of central nervous system sensitization and has been also associated with peripheral nerve damage (e.g., neuropathic pain) [39]. It is possible that patients with migraine exhibiting cutaneous allodynia could also fulfill a neuropathic‐like pain diagnosis. Again, S‐LANSS was not associated with CPM activity in any migraine group.

Although this is the first study investigating the role of clinical migraine features and nociceptive pain processing outcomes in CPM in women with episodic and chronic migraine, some limitations should be considered. First, the cross‐sectional nature of the study did not permit determining a cause‐and‐effect direction of current findings. Second, we just included women with migraine. Given the increased risk of migraine (and chronic pain as well in general) in females and the biological sex differences in pain processing, it would be expected that women might have a different CPM response than men. Hence, our results should not be extrapolated to men. Third, the absence of a pain‐free control group does not permit confirming or refusing the presence of a real CPM deficit in women with migraine. Fourth, although we used the cold‐pressor test as the CPM paradigm since it has shown good reliability [29], it seems that different CPM paradigms lead to different results [8, 9]. This paradigm dependence could be related to the fact that CPM is used as a proxy to evaluate descending pain inhibitory mechanisms where several areas, such as the periaqueductal gray substance, the rostral ventromedial medulla, or the somatosensory cortex, are involved. Thus, it would be possible that different paradigms lead to different pain inhibition responses. Accordingly, it is possible that the duration of immersion (one minute) did not last long enough or that water temperature (10°C) was not enough painful to activate properly the descending inhibitory pathways in women with migraine. No study has compared differences in descending pain inhibition according to the CPM paradigm in the same population. Fifth, prophylactic medication intake was not modified, which may introduce some bias in pain processing outcomes. It should be noted that no differences in either prophylactic or symptomatic medication consumption were observed between women with episodic or chronic migraine. Finally, some features able to further enhance signs and symptoms related to sensitization, such as concomitant neck pain, were not assessed [40].

## 5. Conclusions

This exploratory study found similar CPM efficiency (lack of) to both mechanical and thermal sensitivity between women with episodic and chronic migraine. In addition, the clinical features and the presence of sensitization‐associated or neuropathic‐like pain symptoms did not modulate CPM activity in women with episodic and chronic migraine.

## Author Contributions

All the authors cited in the manuscript had substantial contributions to the concept and design, the execution of the work, or the analysis and interpretation of data and drafting or revising the manuscript. Ana I. de‐la‐Llave‐Rincón: methodology, validation, data curation, writing–original draft, and writing–review and editing. Silvia Ambite‐Quesada: methodology, conceptualization, validation, data curation, writing–original draft, and writing–review and editing. Juan C. Pacho‐Hernández: validation, statistical analysis, data curation, writing–original draft, and writing–review and editing. Angela Tejera‐Alonso: validation, methodology, data curation, writing–original draft, and writing–review and editing. Cristina Gómez‐Calero: conceptualization, methodology, data curation, writing–original draft, and writing–review and editing. César Fernández‐de‐las‐Peñas: conceptualization, methodology, visualization, validation, data curation, writing–original, and writing–review and editing. Beatriz Alvarez‐Mariño: methodology, validation, data curation, writing–original draft, and writing–review and editing. Lars Arendt‐Nielsen: validation, data curation, writing–original draft, and writing–review and editing. Margarita Cigarán‐Méndez: conceptualization, methodology, validation, data curation, writing–original draft, and writing–review and editing.

## Funding

The project has been funded by the Ilustre Colegio Profesional de Fisioterapeutas de la Comunidad de Madrid y el Colegio Oficial de Farmacéuticos de Madrid in a competitive call, through the Call for Multidisciplinary Research Grants in Pain and Physiotherapy, on the occasion of the Pain and Physiotherapy 2024 Congress.

## Disclosure

Both sponsors had no role in the design, collection, management, analysis, or interpretation of the data, draft, review, or approval of the manuscript or its content. The authors were responsible for the decision to submit the manuscript for publication, and the sponsor did not participate in this decision. All authors have read and approved the final version of the manuscript.

## Conflicts of Interest

The authors declare no conflicts of interest.

## Data Availability

The data that support the findings of this study are available on request from the corresponding author.

## References

[1] GBD 2017 US Neurological Disorders Collaborators , Feigin V. L. , Vos T. et al., Burden of Neurological Disorders Across the US From 1990-2017, JAMA Neurology. (2021) 78, 165–176. 33136137 10.1001/jamaneurol.2020.4152 PMC7607495

[2] Ashina M. , Katsarava Z. , Do T. P. et al., Migraine: Epidemiology and Systems of Care, Lancet. (2021) 397, no. 10283, 1485–1495, 10.1016/s0140-6736(20)32160-7. 33773613

[3] Safiri S. , Pourfathi H. , Eagan A. et al., Global, Regional, and National Burden of Migraine in 204 Countries and Territories, 1990 to 2019, Pain. (2022) 163, no. 2, e293–e309, 10.1097/j.pain.0000000000002275. 34001771

[4] Coppola G. and Ambrosini A. , What Has Neurophysiology Revealed About Migraine and Chronic Migraine?, Handbook of Clinical Neurology. (2023) 198, 117–133, 10.1016/B978-0-12-823356-6.00003-2. 38043957

[5] Yarnitsky D. , Arendt-Nielsen L. , Bouhassira D. et al., Recommendations on Terminology and Practice of Psychophysical DNIC Testing, European Journal of Pain. (2010) 14, no. 4, 10.1016/j.ejpain.2010.02.004, 2-s2.0-77949916340. 20227310

[6] Nahman-Averbuch H. , Shefi T. , Schneider V. J. et al., Quantitative Sensory Testing in Patients With Migraine: A Systematic Review and Meta-Analysis, Pain. (2018) 159, no. 7, 1202–1223, 10.1097/j.pain.0000000000001231, 2-s2.0-85053640330. 29781957

[7] Fernández-de-las-Peñas C. , Navarro-Santana M. J. , Curiel-Montero F. , Plaza-Manzano G. , Alburquerque-Sendín F. , and Rodrigues-de-Souza D. P. , Localized and Widespread Pressure Pain Hypersensitivity in Patients With Episodic or Chronic Migraine: A Systematic Review and Meta-Analysis, Cephalalgia. (2022) 42, no. 9, 966–980, 10.1177/03331024221084217. 35332797

[8] Nahman-Averbuch H. , Callahan D. , Darken R. , and Haroutounian S. , Harnessing the Conditioned Pain Modulation Response in Migraine Diagnosis, Outcome Prediction, and Treatment-A Narrative Review, Headache. (2023) 63, no. 8, 1167–1177, 10.1111/head.14601. 37522350

[9] van Welie F. C. , Dahan A. , van Velzen M. , and Terwindt G. M. , Pain Profiling in Migraine: A Systematic Review of Quantitative Sensory Testing (QST), Conditioned Pain Modulation (CPM), and Corneal Confocal Microscopy (CCM), The Journal of Headache and Pain. (2024) 25, no. 1, 10.1186/s10194-024-01932-x. PMC11660458 39701963

[10] Scerbo T. , Colasurdo J. , Dunn S. , Unger J. , Nijs J. , and Cook C. , Measurement Properties of the Central Sensitization Inventory: A Systematic Review, Pain Practice. (2018) 18, no. 4, 544–554, 10.1111/papr.12636, 2-s2.0-85034633514. 28851012

[11] Bennett M. I. , Smith B. H. , Torrance N. , and Potter J. , The S-LANSS Score for Identifying Pain of Predominantly Neuropathic Origin: Validation for Use in Clinical and Postal Research, Journal of Pain. (2005) 6, no. 3, 149–158, 10.1016/j.jpain.2004.11.007, 2-s2.0-14944345606. 15772908

[12] Suzuki K. , Suzuki S. , Shiina T. , Kobayashi S. , and Hirata K. , Central Sensitization in Migraine: A Narrative Review, Journal of Pain Research. (2022) 15, 2673–2682, 10.2147/jpr.s329280. 36101891 PMC9464439

[13] Chakravarty A. and Sen A. , Migraine, Neuropathic Pain and Nociceptive Pain: Towards a Unifying Concept, Medical Hypotheses. (2010) 74, no. 2, 225–231, 10.1016/j.mehy.2009.08.034, 2-s2.0-72449159573. 19765908

[14] Singh S. , Kopruszinski C. M. , Watanabe M. , Dodick D. W. , Navratilova E. , and Porreca F. , Female-Selective Mechanisms Promoting Migraine, The Journal of Headache and Pain. (2024) 25, no. 1, 10.1186/s10194-024-01771-w. PMC11040950 38658853

[15] Verhagen I. E. , van der Arend B. W. , van Casteren D. S. , le Cessie S. , MaassenVanDenBrink A. , and Terwindt G. M. , Sex Differences in Migraine Attack Characteristics: A Longitudinal E-Diary Study, Headache. (2023) 63, no. 3, 333–341, 10.1111/head.14488. 36942410

[16] von Elm E. , Altman D. G. , Egger M. et al., The Strengthening the Reporting of Observational Studies in Epidemiology (STROBE) Statement: Guidelines for Reporting Observational Studies, Epidemiology. (2007) 18, no. 6, 800–804, 10.1097/ede.0b013e3181577654, 2-s2.0-37349032506. 18049194

[17] ICHD-III Headache Classification Subcommittee of the International Headache Society, The International Classification of Headache Disorders. (2018) 38, S1–S211.

[18] Phillip D. , Lyngberg A. C. , and Jensen R. , Assessment of Headache Diagnosis: A Comparative Population Study of a Clinical Interview With a Diagnostic Headache Diary, Cephalalgia. (2007) 27, 1–8, 10.1111/j.1468-2982.2007.01239.x, 2-s2.0-33845886684. 17212676

[19] Stewart W. F. , Lipton R. B. , Whyte J. et al., An International Study to Assess Reliability of the Migraine Disability Assessment (MIDAS) Score, Neurology. (1999) 53, no. 5, 988–994, 10.1212/wnl.53.5.988. 10496257

[20] Stewart W. F. , Lipton R. B. , Kolodner K. , Sawyer J. , Lee C. , and Liberman J. N. , Validity of the Migraine Disability Assessment (MIDAS) Score in Comparison to a Diary-Based Measure in a Population Sample of Migraine Sufferers, Pain. (2000) 88, no. 1, 41–52, 10.1016/s0304-3959(00)00305-5, 2-s2.0-0034307136. 11098098

[21] Rodríguez-Almagro D. , Achalandabaso A. , Rus A. , Obrero-Gaitán E. , Zagalaz-Anula N. , and Lomas-Vega R. , Validation of the Spanish Version of the Migraine Disability Assessment Questionnaire (MIDAS) in University Students With Migraine, BMC Neurology. (2020) 20, no. 1, 10.1186/s12883-020-01646-y. PMC7038557 32093620

[22] Yang M. , Rendas-Baum R. , Varon S. F. , and Kosinski M. , Validation of the Headache Impact Test (HIT-6™) Across Episodic and Chronic Migraine, Cephalalgia. (2011) 31, no. 3, 357–367, 10.1177/0333102410379890, 2-s2.0-79953197234. 20819842 PMC3057423

[23] Martin M. , Blaisdell B. , Kwong J. W. , and Bjorner J. B. , The Short-Form Headache Impact Test (HIT-6) Was Psychometrically Equivalent in Nine Languages, Journal of Clinical Epidemiology. (2004) 57, no. 12, 1271–1278, 10.1016/j.jclinepi.2004.05.004, 2-s2.0-11144254063. 15617953

[24] Cuesta-Vargas A. I. , Roldan-Jimenez C. , Neblett R. , and Gatchel R. J. , Cross-Cultural Adaptation and Validity of the Spanish Central Sensitization Inventory, SpringerPlus. (2016) 5, no. 1, 10.1186/s40064-016-3515-4, 2-s2.0-84991804333. PMC5074937 27818875

[25] Neblett R. , Cohen H. , Choi Y. et al., The Central Sensitization Inventory (CSI): Establishing Clinically Significant Values for Identifying Central Sensitivity Syndromes in an Outpatient Chronic Pain Sample, Journal of Pain. (2013) 14, no. 5, 438–445, 10.1016/j.jpain.2012.11.012, 2-s2.0-84876966634. 23490634 PMC3644381

[26] Cuesta-Vargas A. I. , Neblett R. , Chiarotto A. et al., Dimensionality and Reliability of the Central Sensitization Inventory in a Pooled Multi-Country Sample, Journal of Pain. (2018) 19, no. 3, 317–329, 10.1016/j.jpain.2017.11.006, 2-s2.0-85041536833. 29198933

[27] López-de-Uralde-Villanueva I. , Gil-Martínez A. , Candelas-Fernández P. , de Andrés-Ares J. , Beltrán-Alacreu H. , and La Touche R. , Validity and Reliability of the Spanish-Language Version of the Self-Administered Leeds Assessment of Neuropathic Symptoms and Signs (S-LANSS) Pain Scale, Neurologia. (2018) 33, no. 8, 505–514, 10.1016/j.nrleng.2016.10.003. 27939112

[28] Reezigt R. R. , Kielstra S. C. , Coppieters M. W. , and Scholten-Peeters G. G. M. , No Relevant Differences in Conditioned Pain Modulation Effects Between Parallel and Sequential Test Design. A Cross-Sectional Observational Study, PeerJ. (2021) 9, 10.7717/peerj.12330. PMC8679953 35003911

[29] Nuwailati R. , Curatolo M. , LeResche L. , Ramsay D. S. , Spiekerman C. , and Drangsholt M. , Reliability of the Conditioned Pain Modulation Paradigm Across Three Anatomical Sites, Scandinavian Journal of Pain. (2020) 20, no. 2, 283–296, 10.1515/sjpain-2019-0080. 31812949

[30] Rolke R. , Baron R. , Maier C. et al., Quantitative Sensory Testing in the German Research Network on Neuropathic Pain (DFNS): Standardized Protocol and Reference Values, Pain. (2006) 123, no. 3, 231–243, 10.1016/j.pain.2006.01.041, 2-s2.0-33745294656. 16697110

[31] Billens A. , Dhondt E. , Dierickx E. et al., Attentional Focus but Not Distraction or Expectations Influence Conditioned Pain Modulation in Healthy Adults, European Journal of Pain. (2025) 29, no. 6, 10.1002/ejp.70058. 40542602

[32] Yarnitsky D. , Bouhassira D. , Drewes A. M. et al., Recommendations on Practice of Conditioned Pain Modulation (CPM) Testing, European Journal of Pain. (2015) 19, no. 6, 805–806, 10.1002/ejp.605, 2-s2.0-84929072254. 25330039

[33] Richardson J. T. E. , Eta Squared and Partial Eta Squared as Measures of Effect Size in Educational Research, Educational Research Review. (2011) 6, no. 2, 135–147, 10.1016/j.edurev.2010.12.001, 2-s2.0-79957645252.

[34] Cnockaert E. , Meeus M. , Cagnie B. et al., Human Assumed Central Sensitization in Interictal Migraine: A Systematic Review and Meta-Analysis, Neurological Sciences. (2026) 47, no. 3, 10.1007/s10072-026-08825-8. 41667877

[35] Barone M. , Imaz F. , De la Torre Canales G. , Venosta M. , Dri J. , and Intelangelo L. , Somatosensory and Psychosocial Profile of Migraine Patients: A Cross-Sectional Study, Musculoskeletal Science & Practice. (2024) 70, 10.1016/j.msksp.2024.102924. 38422705

[36] Hübscher M. , Moloney N. , Leaver A. , Rebbeck T. , McAuley J. H. , and Refshauge K. M. , Relationship Between Quantitative Sensory Testing and Pain or Disability in People With Spinal Pain-A Systematic Review and Meta-Analysis, Pain. (2013) 154, no. 9, 1497–1504, 10.1016/j.pain.2013.05.031, 2-s2.0-84881670360. 23711482

[37] Spierings E. L. , Headache Continuum: Concept and Supporting Evidence From Recent Study of Chronic Daily Headache, The Clinical Journal of Pain. (2001) 17, no. 4, 337–340, 10.1097/00002508-200112000-00008, 2-s2.0-0035215701. 11783814

[38] Mínguez-Olaondo A. , Quintas S. , Morollón Sánchez-Mateos N. et al., Cutaneous Allodynia in Migraine: A Narrative Review, Frontiers in Neurology. (2022) 12, 10.3389/fneur.2021.831035. PMC8830422 35153995

[39] Jensen T. S. and Finnerup N. B. , Allodynia and Hyperalgesia in Neuropathic Pain: Clinical Manifestations and Mechanisms, Lancet Neurology. (2014) 13, no. 9, 924–935, 10.1016/s1474-4422(14)70102-4, 2-s2.0-84906076303. 25142459

[40] Di Antonio S. , Arendt-Nielsen L. , Ponzano M. et al., Migraine Patients With and Without Neck Pain: Differences in Clinical Characteristics, Sensitization, Musculoskeletal Impairments, and Psychological Burden, Musculoskeletal Science & Practice. (2023) 66, 10.1016/j.msksp.2023.102800. 37344290

